# Robust biomarker discovery for hepatocellular carcinoma from high-throughput data by multiple feature selection methods

**DOI:** 10.1186/s12920-021-00957-4

**Published:** 2021-08-25

**Authors:** Zishuang Zhang, Zhi-Ping Liu

**Affiliations:** 1 Department of Biomedical Engineering, School of Control Science and Engineering, Shandong University, Jinan, 250061 Shandong China; 2 Center for Intelligent Medicine, Shandong University, Jinan, 250061 Shandong China

**Keywords:** Biomarker discovery, Omics data, Feature selection, Akaike information criterion, Hepatocellular carcinoma

## Abstract

**Background:**

Hepatocellular carcinoma (HCC) is one of the most common cancers. The discovery of specific genes severing as biomarkers is of paramount significance for cancer diagnosis and prognosis. The high-throughput omics data generated by the cancer genome atlas (TCGA) consortium provides a valuable resource for the discovery of HCC biomarker genes. Numerous methods have been proposed to select cancer biomarkers. However, these methods have not investigated the robustness of identification with different feature selection techniques.

**Methods:**

We use six different recursive feature elimination methods to select the gene signiatures of HCC from TCGA liver cancer data. The genes shared in the six selected subsets are proposed as robust biomarkers. Akaike information criterion (AIC) is employed to explain the optimization process of feature selection, which provides a statistical interpretation for the feature selection in machine learning methods. And we use several methods to validate the screened biomarkers.

**Results:**

In this paper, we propose a robust method for discovering biomarker genes for HCC from gene expression data. Specifically, we implement recursive feature elimination cross-validation (RFE-CV) methods based on six different classication algorithms. The overlaps in the discovered gene sets via different methods are referred as the identified biomarkers. We give an interpretation of the feature selection process based on machine learning using AIC in statistics. Furthermore, the features selected by the backward logistic stepwise regression via AIC minimum theory are completely contained in the identified biomarkers. Through the classification results, the superiority of interpretable robust biomarker discovery method is verified.

**Conclusions:**

It is found that overlaps among gene subsets contain different quantitative features selected by the RFE-CV of 6 classifiers. The AIC values in the model selection provide a theoretical foundation for the feature selection process of biomarker discovery via machine learning. What’s more, genes containing in more optimally selected subsets make better biological sense and implication. The quality of feature selection is improved by the intersections of biomarkers selected from different classifiers. This is a general method suitable for screening biomarkers of complex diseases from high-throughput data.

**Supplementary Information:**

The online version contains supplementary material available at 10.1186/s12920-021-00957-4.

## Background

The number of cancer deaths worldwide indicates HCC is the second leading cause in recent years [1]. Despite the practice of surveillance program, most of HCC patients are often examined in advanced stage [2]. Studies have shown liver cancer patients can significantly benefit from early screening [3]. Using effective molecular biomarkers is one of the most efficient way of realizing early cancer diagnosis. The availability of high-throughput omics data provides unprecedented opportunity and challenge for discoverying diagnostic biomarkers for HCC. For instance, the cohort study of TCGA provides amount of valuable data resources for the searching of cancer biomarkers [4].

So far, a number of methods of feature selection have been proposed to identify biomarkers from high-throughput data [5]. Joint with machine-learning-based classification algorithms, feature selection is a very useful strategy for biomarker discovery from ultra-dimensional omics data [6]. Usually, different feature selection methods may produce different feature ranking. Due to the high dimensionality of omics data, feature rankings have more possibilities [7]. Therefore, it is of great significance to realize the reproducibility of biomarkers and the robustness of biomarker discovery. Among the current feature selection methods, more attention is paid to whether the selected biomarkers can achieve a good classification performance. For example, the reference [8] has compared the classification performance of 10 kinds of machine learning algorithms. The applications of 6 kinds of machine learning methods for omics data have been implemented previously [9]. In addition, for the feature selection method of TCGA data, the reference [10] used network smoothing technology combined with PCA to select features. The reference [11] combined multiple levels of TCGA data to find key regulators and pathways between normal and tumor samples. A comprehensive feature selection strategy based on fuzzy rules has been experimented on TCGA data [12]. In order to realize the stability and reproducibility of biomarkers, a method combining individual signatures was proposed to improve the stability of feature selection [7]. However, the stability and reproducibility of biomarkers are still needed to be emphasized and strengthened in biomarker identification.

In this paper, we propose a robust method for discovering biomarker genes from transcriptomic data. Specifically, we implement RFE-CV methods based on 6 different classification algorithms, i.e., Adaboost, K-nearest neighbor (KNN), naïve Bayes (NB), neural network (NN), random forest (RF) and support vector machine (SVM). We find their intersections by comparing the subsets of features selected by different classifiers. The repeatability and stability of biomarker discovery can be achieved by using the genes in the overlapping part. Feature selection by machine learning is often regarded as a black-box predictive model. In Tansey’s work, the holdout randomization test is proposed to explain the black box statistically [13]. As we all known, Akaike information criterion (AIC) is very widely used in the selection of statistical models [14]. Inspired by the studies of Tansey and Akaike, we propose to explain RFE-CV with AIC in statistics. For shedding light to the black box of feature selection, we also introduce backward logistic stepwise regression for comparing and verifying the machine-learning-based feature selection process.

## Methods

**Fig. 1:**
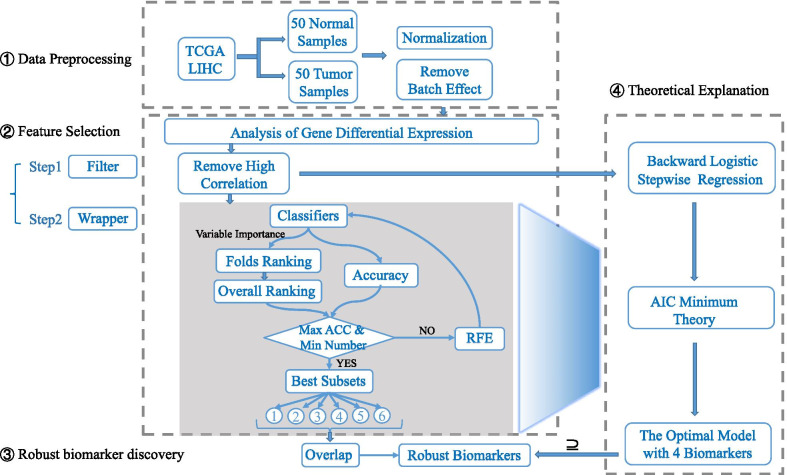
The framework of robust biomarker discovery for HCC

Figure 1 illustrates the framework of identifying cancer biomarkers from TCGA Liver Hepatocellular Carcinoma (LIHC) transcriptomic data, in which the four specific steps are contained.

### Data preprocessing

We download the raw RNA sequencing (RNA-seq) data of HCC from Genomic Data Commons (GDC) using TCGA-Assembler 2.0 [15]. TCGA has sequenced more than 30 types of cancer and aims to provide a valuable data resource for the discovery of biomarkers [4]. For a proof-of-concept study, we use the RNA-seq data of HCC from the TCGA database which contains 20,530 genes and 423 samples, including 50 normal samples and 373 tumor samples. To achieve a balanced dataset in machine learning, we select 50 positive samples and their corresponding 50 negative samples. In other words, a pair of positive and negative samples are from the same donor’s cancerous tissue and adjacent tissue respectively. We normalize the raw RNA-seq data using the median of ratios rule of DESeq2 [16] and remove the batch effects as a covariate. The data and code used in this paper are available at: http://www.github.com/zpliulab/RobMarker.

### Feature selection

Filter, wrapper and embedded techniques are three major types of feature selection methods [17]. In this work, the process of selecting biomarkers mainly consists of two substeps. The first is a filter and the second is a wrapper. In the filtering, we firstly select the genes with differential expression. After data normalization, the differentially expressed genes which meet the threshold requirements form the candidate pool of biomarkers [15]. We set the threshold condition as false discovery rate (FDR) < 0.01 (1%), *P* value < 0.01 and Fold Change > 3. Secondly, we remove one of the redundant features that have a high correlation value with each other. For two genes, if the Pearson’s correlation coefficient between them is greater than 0.65, the gene with the higher mean absolute value is deleted [9].

Wrapper-based feature selection is the focus of our approach. Wrapper adopts the recursive feature selection method based on cross-validation to further select biomarkers from the gene pool which is obtained in the previous step. Here, 6 classical classification algorithms are tested, i.e., Adaboost, KNN, NB, NN, RF, SVM, which are combined with feature selection respectively. Each method is recorded to select the feature subset with the best classification accuracy and the least number of features [18].

### Recursive feature elimination based on cross-validation (RFE-CV)

It has been found that biomarkers selected by RFE-CV have better classification performance than those selected by RFE [19]. For details, the process of RFE-CV can be divided into two procedures [20]. In the first, we apply tenfold cross-validation to get the feature importance ranking. For each feature, we summarize its importance score in every folded dataset to obtain an overall ranking. In the second, the features at the last places in the overall ranking are gradually deleted, until all the features are removed. The best classification subset is determined at the end of the algorithm. In this study, we use the RFE with tenfold cross-validation, so that each feature gets 10 importance scores. We take an average of them for a consensus ranking. Each time a feature is deleted, the classification accuracy of the model is calculated accordingly. The subset with the highest classification accuracy and the least number of features is regarded as the best feature subset. For each feature, we record its importance in each fold to track the change of importance over different folds. Thus, RFE-CV provides more probabilistic estimates of the importance of predictive variables than ranking based only on a single dataset [18].

We find the measure of feature importance by classifiers can directly affect the ranking of features. NN calculates the feature importance based on the connection weights between neurons in hidden layers. It divides the hidden-output connection weight of each hidden neuron into components related to each input neuron. The importance of its characteristics is based on a product that is the absolute value of the hidden-output layer connection weight multiplied by the absolute value of the hidden-input layer connection weight. For the importance of a feature, the sum of its proportion in each neuron is calculated based on the product. Then we calculate the ratio of all the features to the sum of their weights in each neuron [21]. In general, NN determines the relative importance of a feature by identifying all weighted connections between the nodes of interest [21]. We set the number of hidden neurons of NN classifier as 8 and the maximum number of feedback iterations as 30 [22]. In RF, we set the number of decision trees to be 500 [23]. The calculation of feature importance is based on the average value of the difference between the two out-of-pocket error rates of each decision tree [20]. After adding noise to a particular feature, the features that make the accuracy more affected, and often more significant. The argument is that if a feature is important, then the change will greatly affect the test error. If the test error does not change much, then the feature is not important. Some methods calculate the importance of features according to their contributions to the classification performance. For instance, Adaboost, KNN, NB and SVM rank the importance of features according to the AUC values contributed by each feature. Although these four classifiers rank the features according to the classification accuracy obtained by training the model with single feature, the models established by different classifiers for the same feature are different because of different calculation rules. We set the number of weak decision tree classifiers in Adaboost to 10 [24], the parameter k in KNN to 3 [25], and the kernel function in SVM to be linear [26]. NB classifier has no predetermined parameters [27].

### Robust biomarker discovery

For the selection of biomarkers, the repeatability of biomarkers is as important as the classification accuracy of the constructed models. The feature selection method based on wrapper is a strategy guided by machine learning algorithm [28]. Machine learning algorithm is a black box, and we don’t know how it calculates the importance of features. When a feature is calculated using different importance calculations and shows good importance in different ways, we acknowledge that it is a feature that makes sense in the model. If it is only important in a particular method, then we think its importance is not universal. It is more likely to be only related to the computational process of the method and not to the nature of the model. The aim here is to establish a method for robust biomarker discovery. To achieve the reproducibility of biomarkers, we regard the genes with two or more occurrences in the 6 selected-feature subsets as identified biomarkers. We calculate the number of intersection features between the two subsets and perform a hypergeometric test to calculate the significance *P* value of overlapping. Then we analyze the biomarkers that appear in different subsets several times to verify the effectiveness of our method. The more times a feature is selected in different ways, the better its repeatability. We train the model with genes that appear more than four times and then make predictions on independent datasets.

### Backward logistic stepwise regression with AIC

Typically, the feature selection procedure of RFE is a black box. To shed light on the black box, we propose a theoretical explanation of the selection process. We employ AIC value to explain the feature selection process in machine learning and use a backward logistic stepwise regression to explain the results of feature selection.

Stepwise regression is one of the main methods for model selection which has relatively sufficient theoretical basis [29]. By recording the process of feature selection, each step of RFE-CV process is measured by AIC. In this study, the stepwise regression via AIC minimum theory is applied to the candidate pool to select the model with the minimum AIC value.

In order to introduce the maximum likelihood method into the multi-model selection problem, Akaike proposed AIC which is suitable for a wide range of problem. It makes us use the backward stepwise regression method combined with AIC [29]. The problem we interested is a binary classification, so we choose logistic regression for an easy explanation. Logistic regression uses the method of maximizing likelihood estimation and gradient descent method to solve parameters to achieve the purpose of data dichotomy. The goal is to find the best fitting model to describe the relationship between dependent variables and a set of independent (predicted or interpreted) variables [30].

Firstly, we put all features in the candidate set into the model. Secondly, logistic regression is performed on these features [31]. The algorithm tries to remove one of the independent variables from the model to see if there is a significant change in the AIC values. And then it removes the variable that minimizes AIC. This process is repeated until no arguments meet the elimination criteria. Our goal is to find a model with the smallest AIC value.

AIC is widely used in the model selection in statistics [14]. When the goodness of fit of the model is the same degree, the model with fewer variables is preferred [14]. The parsimony principle of model selection is similar to that of RFE-CV. For completeness, we make a brief introduction of the calculation process of AIC. More detailed calculation steps refer to the supplementary material.

Suppose that a random variable $Y$ has a probability density function $f(y|\theta )$, and $\theta$ is the parameter vector. The likelihood function of $\theta$ is defined as $L(\theta ) = f(y_{1} |\theta )f(y_{2} |\theta ) \ldots f(y_{N} |\theta )$. The $g(y)$ is the probability density function that describes the true distribution of $Y$. Here $\mathop \theta \limits^{ \wedge }$ is considered as the estimate of $\theta$ that maximizes the logarithmic likelihood function $l(\theta ) = \ln L(\theta )$. Because of $l(\theta ) = \sum \ln f(y_{i} |\theta )$, then we can get

1 $$\frac{1}{N}l(\theta ) \to E\ln f(Y|\theta ) = \int {g(y)\ln f(y|\theta )} ,\quad N \to \infty .$$

We introduce $m = \frac{{\max L(\theta_{0} )}}{{\max L(\mathop \theta \limits^{ \wedge } )}}$ by means of the methods in the literature [14]. Then we get

2 $$- 2\ln m = - 2\ln \frac{{\max L(\theta_{0} )}}{{\max L(\mathop \theta \limits^{ \wedge } )}} = \Sigma \left[ {\ln \frac{{f(y|\mathop \theta \limits^{ \wedge } )}}{{f(y|\theta_{0} )}}} \right]^{2} .$$

When $N \to \infty$, $- 2\ln m$ asymptotically obeys the chi-square distribution of t degrees of freedom. The $t$ is the dimension of the parameter vector $\theta$. In other words, it is $E\{ 2[l(\mathop \theta \limits^{ \wedge } ) - l(\theta_{0} )])\} = t$. The formulas are as follows

3 $$\begin{aligned} & 2l(\mathop \theta \limits^{ \wedge } ) = 2\Sigma \ln f(y_{i} |\mathop \theta \limits^{ \wedge } ) = 2N\int {f(y_{i} |\mathop \theta \limits^{ \wedge } )\ln f} (y_{i} |\mathop \theta \limits^{ \wedge } )dy \\ & 2E^{*} l(\theta_{0} ) = 2\Sigma \int {f(x_{i} |\theta_{0} )\ln f} (x_{i} |\theta_{0} )dx = 2N\int {f(x_{i} |\theta_{0} )\ln f} (x_{i} |\theta_{0} )dx. \\ \end{aligned}$$

From Formula (3), we know that the adjacent shape of $2l(\theta )$ at $\theta = \mathop \theta \limits^{ \wedge }$ can be approximated by the adjacent shape of $2E^{*} l(\theta )$ at $\theta = \theta_{0}$. $2l(\theta )$ and $2E^{*} l(\theta )$ are approximated by quadric surfaces with vertices $\mathop \theta \limits^{ \wedge }$ and $\theta_{0}$. That means that $2E^{*} l(\theta_{0} )$ is $t$ higher than $2E^{*} l(\mathop \theta \limits^{ \wedge } )$ on average. So the estimate of $E\{ 2E^{*} l(\mathop \theta \limits^{ \wedge } )\} = 2NE^{*} E\ln f(Y|\mathop \theta \limits^{ \wedge } )$ is $2l(\mathop \theta \limits^{ \wedge } ) - 2t$. Then we can get

4 $$AIC = - 2l(\mathop \theta \limits^{ \wedge } ) + 2t.$$

When there is a big difference between the two models, the first term plays a major role in the difference. When the models are not very different, the second term plays a major role. A more detailed derivation can be found in the Additional file 1.

## Results

### Classification and feature selection

**Fig. 2:**
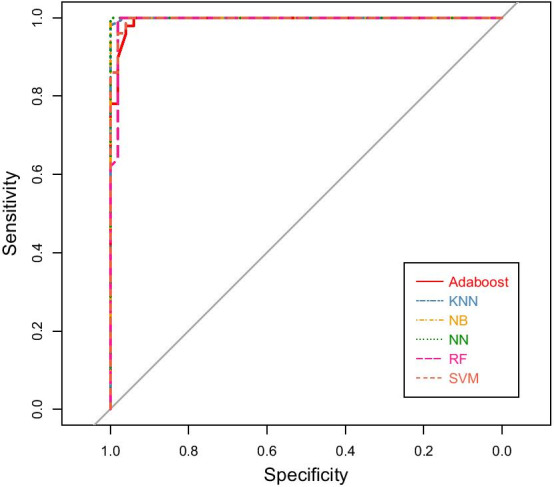
ROC curves corresponding to the best subsets selected by 6 classification algorithms

We obtain the 6 best classification subsets corresponding to the 6 machine learning algorithms. The feature subsets contain the least number of features but can enable the machine learning algorithms to achieve their best classification performances individually. When each classifier reaches its maximum classification accuracy, we obtain its classification performance. Five evaluation parameters, sensitivity (SN), specificity (SP), F1-score, accuracy (ACC) and AUC (area under curve) are used. The ROC curves are shown in Fig. 2. The corresponding evaluation metrics are shown in Table 1. From them, we find that each classifier achieves good classification performance. The classification accuracy of NB and RF reaches 0.99, i.e., only one sample is misclassified. It demonstrates the effectiveness of our method in selecting biomarkers for classifying HCC and control samples.

**Table 1:** The classification performance of the 6 classifiers

Method	# of gene	SN	SP	F1-score	ACC	AUC
Adaboost	21	0.940	1.00	0.969	0.970	0.994
KNN	62	0.960	1.00	0.979	0.980	0.999
NB	12	0.980	1.00	0.989	0.990	1.00
NN	63	0.960	1.00	0.979	0.980	1.00
RF	13	0.980	1.00	0.989	0.990	0.993
SVM	57	0.960	1.00	0.979	0.980	0.996

**Fig. 3:**
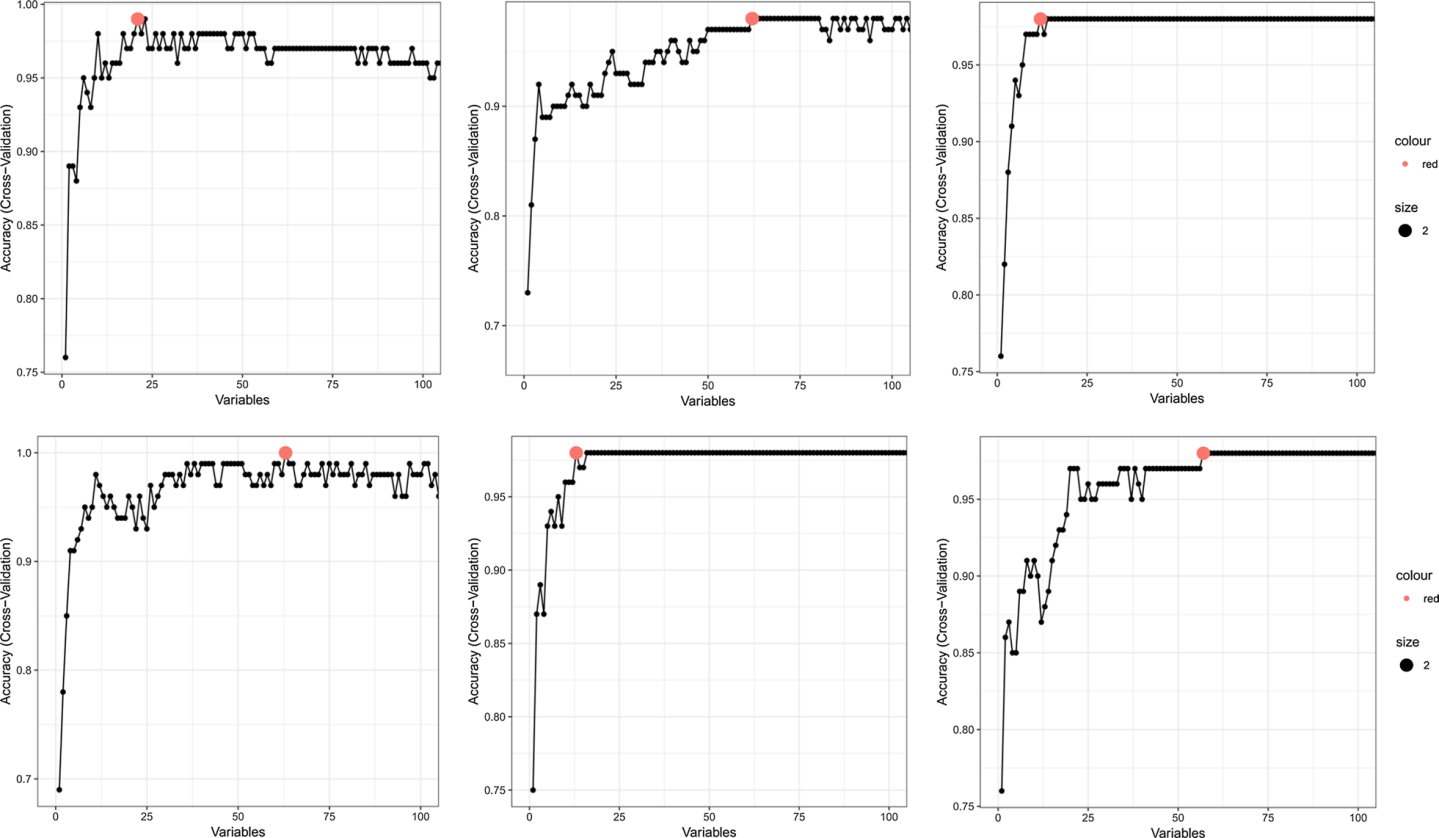
The process of RFE-CV in 6 classifiers. Red point refers to these features with the maximum accuracy

Because of the different ways of calculating feature importance, the 6 RFE-CV methods select the best subset individually. Thus, the selected feature subsets contain different genes. In the feature selection process, we will iteratively obtain a new model after removing a feature. In order to make full use of all samples, we implement tenfold cross-validation classification for the new model. By changing the number of features for a model, different classification accuracy can be obtained. Figure 3 illustrates the correspondence between classification accuracy and feature number. The red points annotate the best feature subset with the highest classification accuracy and the least number of genes. Among them, the subset selected by NB contains the least number of features, namely 12 genes, followed by 13 genes selected by RF classifier.

### Discovery of biomarkers

**Fig. 4:**
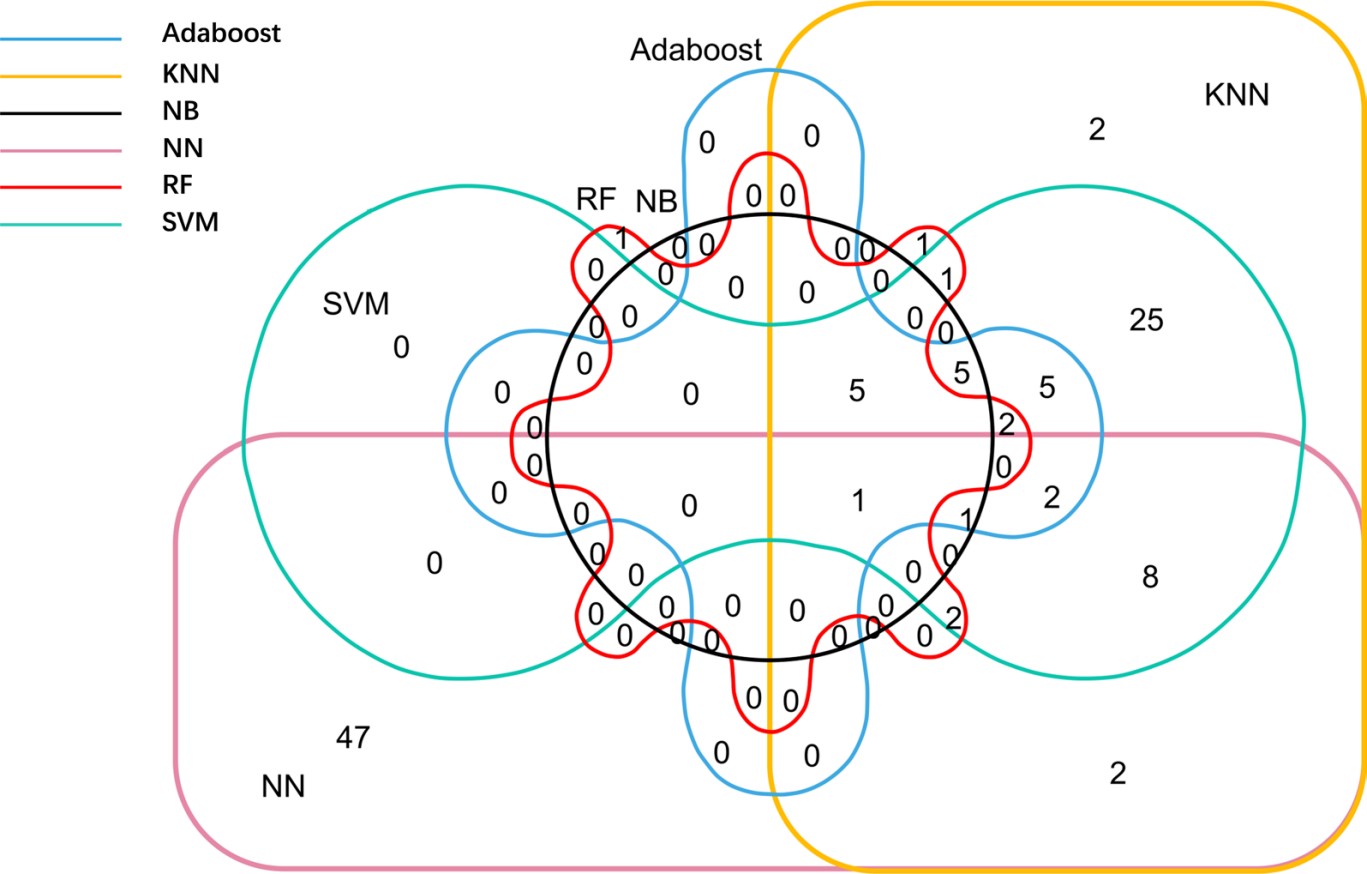
The overlap status of the 6 optimal feature subsets

We compare 6 RFE-CV methods and find a lot of overlaps between the optimal gene subsets. The specific relationship between them is shown in Fig. 4. As described, genes in two or more selected subsets are regarded as the identified biomarkers. A total of 110 genes are selected by the former 6 methods, and 60 genes are contained in the overlaps, which are considered as biomarkers. Among them, 32 genes are contained in 3 or more optimal feature subsets, which are considered to have more functional significance.

Firstly, the 6 optimal subsets have a common biomarker, SKAP1. Also, SKAP1 is the last feature of the stepwise regression deletion, although it is not included in the final four biomarkers identified by stepwise regression. Studies have revealed that SKAP1 is a gene involved in the biological process of immune T cells [32]. It is also shown to be related to the signaling pathways of HCC [33].

Secondly, we find that the four optimal feature subsets selected by Adaboost, KNN, NB and SVM classifiers have inclusion relations. Among them, subsets with a larger number of features contain the subsets with a smaller number of features. The reason is underlying their same feature ranking method. The four classifiers measure the importance of each feature based on the AUC value when each feature is classified separately. By comparing the genes in the four identified optimal feature subsets, we find most of these features rank at the same places in the four classifiers. The important genes identified by different classifiers indicate the effectiveness and consistency of different feature selection strategies.

Moreover, we find that there are a lot of overlaps between the features selected by RF and the features selected by the four classifiers. 6 out of 12 features selected by NB appear in the 13 features selected by RF. The feature subsets selected by NN and KNN have the largest intersection, with 16 features. The overlap between two subsets is shown in Table 2. We employ the hypergeometric distribution test for achieving its statistical significance *P* value [34].

**Table 2:** The number of overlapping features and the corresponding significance *P* values

Overlap	Adaboost	KNN	NB	NN	RF	SVM	SR
Adaboost	21	21	12	4	8	21	2
KNN	< 1e−6	62	12	16	12	57	4
NB	< 1e−6	< 1e−6	12	2	6	12	2
NN	6e-2	< 1e−6	2.1e-1	63	3	14	3
RF	< 1e−6	< 1e−6	< 1e−6	6e-2	13	11	0
SVM	< 1e−6	< 1e−6	< 1e−6	1.43e−5	< 1e−6	57	4
SR	5.36e-4	1.82e-5	9.95e-4	1.3e-3	1	1.55e-5	4

Some biomarkers that appear more frequently in the selected subsets are considered as important feature genes, which should be explored their biological functional significance in later sections.

### Statistical interpretation of feature selection in machine learning

In the candidate biomarker pool, we perform a backward logistic stepwise regression on 886 differentially expressed genes [35]. The AIC values are used to measure the quality of the model iteratively. After putting all the features in the model, we remove sequentially the ones that reduce the AIC value with the fastest speed. At the end of the algorithm, the final best model has the smallest AIC value. It selects four feature genes, namely COL9A1, PHOSPHO1, EGFL6 and OXT. All the four features are included in the biomarker set selected by the former 6 REF-CV methods. Since the stepwise regression selects very few features, we analyze it from another perspective. We find that the relatively important features deleted near the end of stepwise regression are actually included in the selected biomarker set. Moreover, the *P* values of these features are very small. It indidates they are significantly differentially expressed genes. From the beginning of stepwise regression, there is a tendency for the latter deletion feature to have a smaller *P* value. Table 3 lists the last-deleted five features in the stepwise regression process, and we can determine that they are indeed included in our biomarker collection.

**Table 3:** The last 5 genes deleted by stepwise regression

Step	Deviance	Resid. Dev	*P* value	AIC
ID2B	7.72e−11	1.91e−09	1.73e−17	18 + 1.91e−09
PMP2	4.55e−10	2.37e−10	1.05e−19	16 + 2.37e−10
MUC6	5.45e−10	2.91e−10	1.59e−07	14 + 2.91e−10
C1QL1	1.03e−09	3.94e−09	1.74e−24	12 + 3.94e−09
SKAP1	1.25e−08	1.64e−08	4.19e−13	10 + 1.64e−08

We also analyze the overlap between the four features selected by the backward logistic stepwise regression and the optimal subsets of each classifier. The four features of the backward logistic stepwise regression are fully contained in the feature subsets of KNN and SVM, and three features are in the subset of NN. The overlap of backward logistic stepwise regression and the other optimal subsets are illustrated in Tables 2 and 3 respectively.

**Fig. 5:**
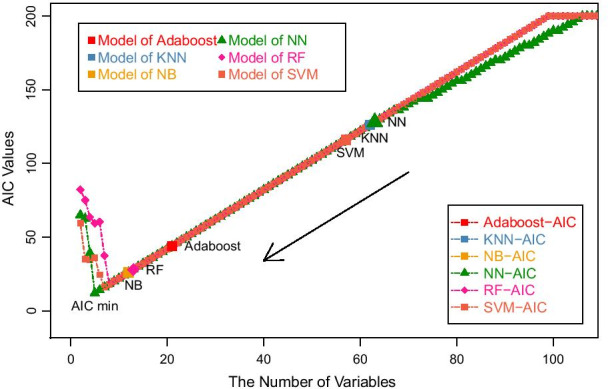
The trend of AIC value in the feature selection process

Synchronization with the iterative elimination of RFE-CV, for each deletion of a feature in the iteration, logistic regression is performed on the new model and its AIC value is calculated correspondingly. By calculating AIC values, we statistically interprete the feature selection process for each classifier. The relationship between AIC values and feature numbers is shown in Fig. 5.

As illustrated in Fig. 5, AIC values gradually decrease with the decreasing number of features. Except the NN classifier, AIC values based on the other five classifiers all decline at a slope of 2. When the number of features is large, the features fit the model roughly the same. The number of features has a large impact on the AIC value. When the AIC value drops to a certain point (< 20), it tends to increase sharply. The turning point has the minimum AIC value. When the number of features in the subset is less than the turning point, the goodness of fit to the model will deteriorate. In this case, the number of features will not be the dominant factor, thus the AIC value will increase.

In Fig. 5, we also annotate the AIC values of models when each classifier identifies its optimal feature subset. NB has the lowest AIC value, followed by RF. Although the classification performances obtained by the models trained by KNN, NN and SVM are good, their corresponding minimum AIC values are relatively large due to the large number of features. Using simple machine learning methods to make feature selection, biomarkers cannot be excellently selected because redundant features may still exist. Therefore, AIC can be combined with the RFE method as a mentor, guiding the feature selection process for robust biomarker discovery.

According to the change trend of AIC value shown in Fig. 5, we theoretically explain the feature selection process. As is known to all, the feature selection process based on AIC is a process in which the goodness of model fit increases and the number of features decreases [14]. Through our method, we prove that the feature selection process of biomarker discovery based on machine learning is also such a process.

### Validation of biomarkers

We verify the effectiveness of the identified biomarkers in three ways, which will also prove the effectiveness of our proposed method. The genes in the intersection are identified as the robust biomarkers. The higher the frequency of gene emergence, the more likely it is to precisely diagnose the disease.

Firstly, we make a statistics of the biological functions of some important genes. Some genes have been verified to be closely associated with the occurrence and development of HCC. Table 4 lists some representative biomarker genes in the overlaps. From the dysfunctions of genes listed in Table 4, we can conclude that the method of identifying biomarkers from overlaps is effective.

**Table 4:** Some genes and their dysfunctions from the interactions of selected feature subsets of different methods

Gene	Subset	Function
SKAP1	6 methods	SKAP1 encodes a T cell adaptor protein and it is involved in HCC signaling pathways [32, 33]
EPHB1	SVM, KNN, NN	Ephrin-B1 participates in the tumor progression through promoting the formation of new vessels of HCC [36]
STC2	NN, SVM, KNN	STC2 is overexpressed in HCC and acts as a potential oncoprotein [37]
CDHR2	NN, SVM, KNN	CDHR2 is highly expressed in HCC para-carcinoma tissue, but is weakly expressed in tumors. It is found to inhibit tumor growth [38]
FAM134B	NN, SVM, KNN	FAM134B works as a tumor inhibitor and inhibits cancer growth in vitro and in vivo [39]
MUC6	RF, NN, SVM, KNN	MUC6 encodes a member of the mucin protein family. It is a biomarker gene of many cancers [40]
PHOSPHO1	Adaboost, NB, NN, SVM, KNN, Stepwise Regression	PHOSPHO1 is associated with hepatitis B [41]
OXT	NN, SVM, KNN, Stepwise Regression	OXT is found to regulate cell proliferation. It is a key differential gene in nonalcoholic fatty liver disease [42]

**Fig. 6:**
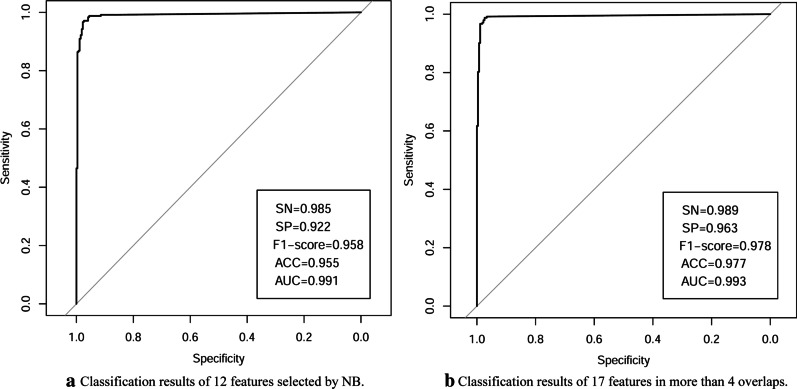
The ROC curves of the trained NB classifier in the independent data GSE25097

Secondly, we validate the features selected by the best-performing classifiers on independent data sets. Based on the former results, we find that NB-RFE-CV achieves the best classification performance. Specifically, it selects the least number of feature subsets and obtain a better AUC value than the other five classifiers. Moreover, the optimal subset corresponding to NB for logistic regression has the minimum AIC value. To verify the results are not caused by overfitting, we use 12 genes selected by NB to classify samples of an independent dataset. We download another HCC dataset from GEO in NCBI with ID GSE25097 [9]. We use the former 100 TCGA samples as the training set and GSE25097 as the testing data set. Figure 6a shows the classification performance of the 12 features. The metrics of SN, SP, F1-score, ACC and AUC are also shown. The results of cross-dataset validation demonstrate that the former good classification ability is not caused by overfitting, and that our proposed method of identifying biomarkers is efficient.

What’s more, it is also necessary to check the model trained by overlap features on independent datasets. There are 17 features that appear more than 4 times in the 6 feature subsets. We use these 17 features to train on the balanced data set of TCGA and test on the independent data set GSE25097. In the same way, NB classifier is selected. Figure 6b shows the classification perforamcne of 17 features.

We compare the two models and find that 17 features in overlap could achieve better classification performance. The latter’s five evaluation indicators are all higher than the former ones. Our proposed method greatly improves the elimination of false positive feature genes. To sum up, the three verifications further justify the effectiveness of our proposed method.

## Discussion

In this paper, we proposed a robust biomarker discovery framework via multiple feature selection methods. The set of differentially expressed genes provides a pool of biomarker candidates. We applied the RFE-CV feature selection methods based on 6 different classification algorithms to select diagnostic biomarkers from the candidate pool. The 6 classifiers respectively get the importance ranking of features and further obtain the best feature subsets. Theoreticallly, AIC was employed to explain the feature selection process of machine learning. In the process of feature reduction, AIC value also decreases, indicating that our feature selection process is statistically interpretable. For the 6 optimal feature subsets selected, we found out their overlaps that serve as robust biomarkers. These discovered biomarkers have been shown to be closely related to the occurrence and development of HCC.

To achieve a statistical explanation of machine learning and feature selection, the genes in the candidate pool are simultaneously regressed step by step according to the AIC minimum theory. We found the subset of genes selected in the adapative regression are completely included in the subset of genes selected by feature selection in machine learning. The consistency further indicates the important features play an important role in training different methods. According to the trend of AIC value changing in the feature selection process, it can be found that the selected features may not be the most concise one only by machine learning. For example, the optimal subsets selected by NN, KNN and SVM contain relatively larger number of biomarker genes. In the model fitting of these selected subsets, the relationship between the AIC goodness and the number of features is not optimal. It is clear that some redundant feature genes still exist in these subsets. Ideally, the model with a minimum AIC value should be selected to ensure the best classification. Although our method can explain the feature selection process based on machine learning, the exploration of explicable feature selection process should go further. We will also continue to study the interpretability of feature selection.

Moreover, we found feature genes contained in more subsets make more biological sense. The quality of feature selection is improved by the intersection of biomarkers. Through the previous experiments, we can prove that our method greatly improves the classification performance, especially the problem of high false positives that the current classification model often achieves.

## Conclusion

In summary, we presented a method for robust biomarker discovery from RNA-seq data based on feature selection with statistical validation. Not only do we provide a statistical interpretation of the machine-learning-based feature selection process, but also the results of gene function enrichment analysis and the validation of independent data sets provide a compelling argument for our approach. More importantly, we proposed a solution to the problem of feature selection instability. Our method is not only applicable to the discovery of HCC biomarkers, but also to the discovery of other disease biomarkers.

## Supplementary Information

**Additional file 1**. Supplementary material for the calculation process of AIC.

## Data Availability

The Cancer Genome Atlas (TCGA) datasets for Liver Hepatocellular Carcinoma [Project ID: TCGA-LIHC, Project Name: Liver Hepatocellular Carcinoma (dbGaP Study Accession: phs000178)] supporting the conclusions of this article are available in the repository in Genomic Data Commons Data Portal at https://portal.gdc.cancer.gov/. The validation data set GSE25097 is available at https://www.ncbi.nlm.nih.gov/. The code used in this paper is available at http://www.github.com/zpliulab/RobMarker.

## Abbreviations

HCC: Hepatocellular carcinoma
TCGA: The cancer genome atlas
RFE: Recursive feature elimination
RFE-CV: Recursive feature elimination cross-validation
AIC: Akaike information criterion
KNN: K-nearest neighbor
NB: Naïve Bayes
NN: Neural network
RF: Random forest
SVM: Support vector machine
LIHC: Liver Hepatocellular Carcinoma
RNA-seq: RNA sequencing
GDC: Genomic Data Commons
FDR: False discovery rate
SN: Sensitivity
SP: Specificity
ACC: Accuracy
AUC: Area under curve
