# Supplementary material for: Robust biomarker discovery for hepatocellular carcinoma from high-throughput data by multiple feature selection methods
Source: BMC Med Genomics. 2021 Aug 25;14(Suppl 1):112. doi: 10.1186/s12920-021-00957-4 (PMC8386074; doi:10.1186/s12920-021-00957-4)
Supplement: Supplementary file 1 — Additional file 1. Supplementary material for the calculation process of AIC. [file 12920_2021_957_MOESM1_ESM.docx]

# Supplementary material for the calculation process of AIC

In the following, we describe the calculation process of AIC in details. Suppose that a random variable $Y$ has a probability density function$f(y|\theta)$, and $\theta$is the parameter vector. When we get a set of independent implementation values$y_{1},y_{2},...,y_{N}$, the likelihood function of $\theta$ is defined as $L(\theta)=f(y_{1}|\theta)f(y_{2}|\theta)...f(y_{N}|\theta)$. The $g(y)$ is the probability density function that describes the true distribution of $Y$. Here $\theta^{\wedge}$ is considered to be the estimate of $\theta$ that maximizes the logarithmic likelihood function$l(\theta)=\ln L(\theta)$. Because of$l(\theta)=\sum\ln f(y_{i}|\theta)$, then we can get

$\frac{1}{N}l(\theta)\to E\ln f(Y|\theta)=\int g(y)\ln f(y|\theta),N\to\infty$. (1)

So $\theta^{\wedge}$ is the largest $\theta$ estimate of $E\ln f(Y|\theta)$. According to Kullback's definition of relative entropy $KL(P//Q)=\int P(x)\log\frac{P(x)}{Q(x)}dx$ [[32](#_ENREF_32)], we plug in the formula to get

$S(g;f(Y|\theta))=\int g(y)\ln\frac{g(y)}{f(y|\theta)}dy=E\ln g(Y)-E\ln f(Y|\theta)$. (2)

According to the non-negative property of Kullback property, there is $\int g(y)\ln\frac{g(y)}{f(y|\theta)}dy\geq0$, if and only if 0 when the distribution of $g(y)$ and $f(y|\theta)$are the same. Therefore, when $E\ln g(Y)-E\ln f(Y|\theta)$ is 0, we maximize $E\ln f(y|\theta)$. In terms of Kullback principle, it is to find $f(y|\theta)$ closest to $g(y)$, which is essentially the same as maximum likelihood.

Use $E^{*}S(g;f(Y|\theta^{\wedge} ))$ as the standard to evaluate $\theta^{\wedge}$. $\theta^{\wedge}$ is a function of our observed $x_{1},x_{2},...,x_{N}$. The $x_{1},x_{2},...,x_{N}$ and $y_{1},y_{2},...,y_{N}$ are independent identity distribution. $E^{*}$ is the mathematical expectation of the distribution of $x_{1},x_{2},...,x_{N}$.When multiple models are compared, $E^{*}E\ln g(Y)$in $E^{*}S(g;f(Y|\theta^{\wedge} ))=E^{*}[E\ln g(Y)-E\ln f(Y|\theta)]$ is a common term that can be omitted. So we just need a good estimate of $E^{*}E\ln f(Y|\theta)$.

We introduce $m=\frac{\max L(\theta_{0})}{\max L(\theta^{\wedge} )}$ by means of the methods in the literature [[14](#_ENREF_14)]. Then we get

$\begin{matrix} -2\ln m=-2\ln\frac{\max L(\theta_{0})}{\max L(\theta^{\wedge} )}=2[l(\theta^{\wedge} )-l(\theta_{0})] \\ =\Sigma[\ln f(y|\theta^{\wedge} )-\ln f(y|\theta_{0})]^{2}=\Sigma[\ln\frac{f(y|\theta^{\wedge} )}{f(y|\theta_{0})}]^{2} \end{matrix}$. (3)

When$N\to\infty$,$-2\ln m$ asymptotically obeys the chi-square distribution of t degrees of freedom. The $t$ is the dimension of the parameter vector$\theta$. In other words, it is$E\{2[l(\theta^{\wedge} )-l(\theta_{0})])\}=t$. The formulas are as follows

$\begin{matrix} 2l(\theta^{\wedge} )=2\Sigma\ln f(y_{i}|\theta^{\wedge} )=2N\int f(y_{i}|\theta^{\wedge} )\ln f(y_{i}|\theta^{\wedge} )dy \\ 2E^{*}l(\theta_{0})=2\Sigma\int f(x_{i}|\theta_{0})\ln f(x_{i}|\theta_{0})dx=2N\int f(x_{i}|\theta_{0})\ln f(x_{i}|\theta_{0})dx \end{matrix}$. (4)

From formula (4), we know that the adjacent shape of $2l(\theta)$ at $\theta=\theta^{\wedge}$ can be approximated by the adjacent shape of $2E^{*}l(\theta)$ at$\theta=\theta_{0}$. $2l(\theta)$ and $2E^{*}l(\theta)$ are approximated by quadric surfaces with vertices $\theta^{\wedge}$ and $\theta_{0}$.That means that $2E^{*}l(\theta_{0})$ is $t$ higher than $2E^{*}l(\theta^{\wedge} )$ on average. So the estimate of $E\text{\{}2E^{*}l(\theta^{\wedge} )\text{\}=}2NE^{*}E\ln f(Y|\theta^{\wedge} )$ is $2l(\theta^{\wedge} )-2t$. Then we can get

$AIC=-2l(\theta^{\wedge} )+2t$. (5)
